# Supplementary material for: Direct and indirect costs attributed to alcohol consumption in Brazil, 2010 to 2018
Source: PLoS One. 2022 Oct 25;17(10):e0270115. doi: 10.1371/journal.pone.0270115 (PMC9595536; doi:10.1371/journal.pone.0270115)
Supplement: S1 Table — Costs attributable to alcohol by type of cost and ICD, Brazil, 2010. (PDF) [file pone.0270115.s001.pdf]

S1 Table: Costs attributable to alcohol by type of cost and ICD, Brazil, 2010

ICD-10	Costs attributed to alcohol - Hospital	Costs attributed to alcohol - Hospital (Lower CI)	Costs attributed to alcohol - Hospital (Upper CI)	Costs attributed to alcohol - Outpatient	Costs attributed to alcohol - Outpatient (Lower CI)	Costs attributed to alcohol - Outpatient (Upper CI)	Costs attributed to alcohol - Absenteeism	Costs attributed to alcohol - Absenteeism (Lower CI)	Costs attributed to alcohol - Absenteeism (Upper CI)
Tuberculosis	2,319,795.39	971,532.70	4,369,432.08	64,059.23	26,828.07	120,658.26	2,962,146.41	1,240,549.97	5,579,327.21
Lower respiratory infections	3,040,966.43	386,172.45	10,501,545.81	12,342.59	1,567.39	42,623.38	80,181.89	10,182.30	276,896.78
Esophageal cancer	1,805,150.51	831,393.95	2,922,362.79	1,863,968.84	858,483.77	3,017,583.94	444,529.44	204,735.89	719,649.86
Liver cancer due to alcohol use	114,723.44	7,244.88	277,055.20	42,197.63	2,664.82	101,906.58	71,799.91	4,534.23	173,395.58
Laryngeal cancer	705,226.24	159,193.46	1,481,199.84	931,854.00	210,351.02	1,957,190.35	263,845.63	59,558.90	554,159.89
Breast cancer	1,828,601.26	1,152,728.41	2,517,886.25	19,857,374.66	12,517,852.01	27,342,544.19	3,161,111.88	1,992,727.21	4,352,682.21
Colon and rectum cancer	2,236,725.51	1,165,098.81	3,354,012.52	4,618,451.03	2,405,727.38	6,925,455.31	906,102.69	471,984.23	1,358,718.24
Lip and oral cavity cancer	3,370,033.38	1,853,726.29	5,073,134.32	3,294,226.84	1,812,028.02	4,959,017.72	988,054.18	543,490.76	1,487,383.36
Nasopharyngeal cancer	202,909.61	184,280.92	221,769.23	801,179.58	727,625.03	875,645.97	173,640.61	157,699.04	189,779.80
Other pharyngeal cancers	979,997.55	540,254.07	1,468,263.51	3,185,230.35	1,755,957.13	4,772,213.45	593,402.39	327,131.49	889,054.34
Hypertensive heart disease	181,891.61	74,346.47	348,341.47	48,952.62	20,008.92	93,749.39	400,090.70	163,533.27	766,215.55
Atrial fibrillation and flutter	159,418.69	94,428.02	230,380.94	2,272.74	91,363.61	106,699.48	52,222.04	30,932.54	75,467.71

ICD-10	Costs attributed to alcohol - Hospital	Costs attributed to alcohol - Hospital (Lower CI)	Costs attributed to alcohol - Hospital (Upper CI)	Costs attributed to alcohol - Outpatient	Costs attributed to alcohol - Outpatient (Lower CI)	Costs attributed to alcohol - Outpatient (Upper CI)	Costs attributed to alcohol - Absenteeism	Costs attributed to alcohol - Absenteeism (Lower CI)	Costs attributed to alcohol - Absenteeism (Upper CI)
Cirrhosis and other chronic liver diseases due to alcohol use	5,004,338.46	2,617,311.68	7,994,936.97	75,572.58	39,525.10	120,734.84	1,249,569.32	653,535.41	1,996,313.41
Pancreatitis	869,155.91	258,885.45	2,235,668.15	192,013.73	57,192.92	493,903.31	260,907.57	77,713.53	671,114.05
Epilepsy	1,007,849.67	464,717.71	1,625,823.63	668,794.05	308,379.76	1,078,872.38	1,148,263.49	529,462.28	1,852,333.71
Transport injuries	6,826,990.97	1,639,615.09	13,152,311.92	6,982.71	1,677.01	13,452.31	8,304.03	1,994.35	15,997.85
Unintentional injuries	10,059,624.14	2,409,492.59	20,837,267.78	11,308.19	2,708.55	23,423.51	8,717.77	2,088.09	18,057.77
Self-harm	214,450.11	32,367.04	488,439.95	206.36	31.15	470.02	3,008.10	454.01	6,851.38
Interpersonal violence	1,803,578.98	407,666.31	3,517,546.31	2,768.56	625.78	5,399.57	12,753.20	2,882.63	24,872.75
Intracerebral hemorrhage - Male	2,818,838.80	965,553.32	4,958,184.26	106,801.44	19,031.67	187,857.93	305,003.76	104,474.72	536,485.05
Intracerebral hemorrhage - Female	947,826.74	- 488,535.09	2,541,153.05	29,976.09	8,229.77	80,366.84	56,944.75	15,633.86	152,670.65
Alcohol use disorders	49,325,074.59			21,223,453.01			22,399,460.06		
TOTAL	95,823,167.98	15,727,474.53	90,116,715.98	57,039,986.85	20,867,858.87	52,319,768.73	35,550,059.83	6,595,298.70	21,697,427.15
