# Supplementary material for: Direct and indirect costs attributed to alcohol consumption in Brazil, 2010 to 2018
Source: PLoS One. 2022 Oct 25;17(10):e0270115. doi: 10.1371/journal.pone.0270115 (PMC9595536; doi:10.1371/journal.pone.0270115)
Supplement: S2 Table — Costs attributable to alcohol by type of cost and ICD, Brazil, 2011. (PDF) [file pone.0270115.s002.pdf]

S2 Table: Costs attributable to alcohol by type of cost and ICD, Brazil, 2011

ICD-10	Costs attributed to alcohol - Hospital	Costs attributed to alcohol - Hospital (Lower CI)	Costs attributed to alcohol - Hospital (Upper CI)	Costs attributed to alcohol - Outpatient	Costs attributed to alcohol - Outpatient (Lower CI)	Costs attributed to alcohol - Outpatient (Upper CI)	Costs attributed to alcohol - Absenteeism	Costs attributed to alcohol - Absenteeism (Lower CI)	Costs attributed to alcohol - Absenteeism (Upper CI)
Tuberculosis	2,784,761.23	1,166,260.87	5,245,214.77	67,380.79	28,219.14	126,914.54	3,076,538.52	1,288,457.50	5,794,789.56
Lower respiratory infections	3,005,353.68	381,649.98	10,378,562.22	11,226.31	1,425.63	38,768.46	76,864.08	9,760.97	265,439.19
Esophageal cancer	1,754,462.83	808,048.84	2,840,304.39	1,959,271.89	902,377.27	3,171,870.29	471,220.04	217,028.71	762,859.33
Liver cancer due to alcohol use	133,872.26	8,454.15	323,299.27	63,971.50	4,039.85	154,490.10	74,726.43	4,719.04	180,463.09
Laryngeal cancer	649,165.50	146,538.65	1,363,454.42	1,123,436.09	253,597.59	2,359,573.78	290,074.67	65,479.68	609,249.24
Breast cancer	1,827,209.47	1,151,851.05	2,515,969.84	22,436,513.69	14,143,710.47	30,893,880.86	3,504,786.91	2,209,375.84	4,825,904.36
Colon and rectum cancer	2,493,017.87	1,298,600.18	3,738,327.80	6,367,899.41	3,317,006.04	9,548,786.49	978,713.69	509,806.92	1,467,599.82
Lip and oral cavity cancer	3,155,066.66	1,735,481.33	4,749,530.69	4,015,112.23	2,208,559.46	6,044,214.21	1,055,288.98	580,474.05	1,588,596.35
Nasopharyngeal cancer	209,187.87	189,982.79	228,631.04	859,233.73	780,349.35	939,096.01	193,684.65	175,902.88	211,686.85
Other pharyngeal cancers	1,081,670.30	596,304.33	1,620,592.85	3,690,308.60	2,034,397.20	5,528,937.77	573,601.23	316,215.49	859,387.62
Hypertensive heart disease	151,217.13	61,808.56	289,596.62	29,819.50	12,188.44	57,107.46	339,804.62	138,891.91	650,761.40

ICD-10	Costs attributed to alcohol - Hospital	Costs attributed to alcohol - Hospital (Lower CI)	Costs attributed to alcohol - Hospital (Upper CI)	Costs attributed to alcohol - Outpatient	Costs attributed to alcohol - Outpatient (Lower CI)	Costs attributed to alcohol - Outpatient (Upper CI)	Costs attributed to alcohol - Absenteeism	Costs attributed to alcohol - Absenteeism (Lower CI)	Costs attributed to alcohol - Absenteeism (Upper CI)
Atrial fibrillation and flutter	166,160.58	98,421.42	240,123.86	1,872.94	1,109.39	2,706.64	52,586.95	31,148.68	75,995.06
chronic liver diseases due to alcohol use	4,680,588.61	2,447,987.75	7,477,713.84	101,776.24	53,229.84	162,597.84	1,161,096.68	607,263.46	1,854,969.42
Pancreatitis	858,099.82	255,592.30	2,207,229.35	132,711.18	39,529.15	341,363.55	218,572.04	65,103.54	562,217.38
Epilepsy	1,041,349.66	480,164.50	1,679,864.51	323,412.89	149,125.11	521,717.01	998,941.66	460,610.25	1,611,453.59
Transport injuries	7,086,738.86	1,701,997.85	13,652,720.57	12,260.96	2,944.67	23,620.94	27,050.78	6,496.69	52,113.79
Unintentional injuries	10,296,298.18	2,466,181.03	21,327,508.80	19,917.25	4,770.60	41,256.11	25,218.06	6,040.26	52,236.09
Self-harm	201,421.93	30,400.69	458,766.46	706.26	106.60	1,608.61	5,439.69	821.01	12,389.65
Interpersonal violence	1,782,875.22	402,986.60	3,477,167.45	5,688.26	1,285.73	11,093.89	44,542.96	10,068.13	86,872.79
Intracerebral hemorrhage - Male	2,636,072.68	902,949.37	4,636,708.59	87,956.74	30,128.34	154,711.12	300,287.22	102,859.13	528,188.90
Intracerebral hemorrhage - Female	788,722.89	216,539.44	2,114,590.68	25,527.66	7,008.47	68,440.44	60,171.93	16,519.86	161,322.82
Alcohol use disorders	45,539,802.30			22,447,272.30			23,121,527.33		
TOTAL	92,323,115.55	16,548,201.71	90,565,878.02	63,783,276.39	23,975,108.35	60,192,756.14	36,650,739.14	6,823,044.02	22,214,496.28
