# Supplementary material for: Direct and indirect costs attributed to alcohol consumption in Brazil, 2010 to 2018
Source: PLoS One. 2022 Oct 25;17(10):e0270115. doi: 10.1371/journal.pone.0270115 (PMC9595536; doi:10.1371/journal.pone.0270115)
Supplement: S3 Table — Costs attributable to alcohol by type of cost and ICD, Brazil, 2012. (PDF) [file pone.0270115.s003.pdf]

S3 Table: Costs attributable to alcohol by type of cost and ICD, Brazil, 2012

ICD-10	Costs attributed to alcohol - Hospital	Costs attributed to alcohol - Hospital (Lower CI)	Costs attributed to alcohol - Hospital (Upper CI)	Costs attributed to alcohol - Outpatient	Costs attributed to alcohol - Outpatient (Lower CI)	Costs attributed to alcohol - Outpatient (Upper CI)	Costs attributed to alcohol - Absenteeism	Costs attributed to alcohol - Absenteeism (Lower CI)	Costs attributed to alcohol - Absenteeism (Upper CI)
Tuberculosis	2,706,063.98	1,133,302.38	5,096,985.20	55,066.36	23,061.85	103,719.80	3,297,980.75	1,381,197.73	6,211,885.31
Lower respiratory infections	2,879,643.52	365,686.05	9,944,440.02	10,529.28	1,337.11	36,361.36	86,825.60	11,025.99	299,839.87
Esophageal cancer	1,751,765.01	806,806.32	2,835,936.88	2,053,985.45	945,999.28	3,325,202.32	515,062.09	237,220.94	833,835.34
Liver cancer due to alcohol use	186,053.97	11,749.47	449,317.23	67,144.23	4,240.22	162,152.19	76,712.29	4,844.45	185,258.91
Laryngeal cancer	612,213.36	138,197.30	1,285,843.14	1,169,474.73	263,990.07	2,456,269.59	311,702.63	70,361.84	654,674.85
Breast cancer	1,962,930.80	1,237,408.15	2,702,850.86	22,246,925.38	14,024,196.26	30,632,827.88	3,993,683.77	2,517,570.59	5,499,089.22
Colon and rectum cancer	2,608,916.20	1,358,971.03	3,912,119.55	6,349,457.73	3,307,399.87	9,521,132.83	1,130,614.46	588,931.25	1,695,377.92
Lip and oral cavity cancer	3,192,199.48	1,755,906.68	4,805,429.18	3,985,867.49	2,192,473.05	6,000,190.17	1,133,208.66	623,334.69	1,705,894.01
Nasopharyngeal cancer	238,998.96	217,056.99	261,212.95	764,009.22	693,867.20	835,020.78	234,016.14	212,531.63	255,766.99
Other pharyngeal cancers	1,193,510.97	657,959.98	1,788,156.12	3,653,178.36	2,013,927.99	5,473,308.05	580,728.37	320,144.55	870,065.73

ICD-10	Costs attributed to alcohol - Hospital	Costs attributed to alcohol - Hospital (Lower CI)	Costs attributed to alcohol - Hospital (Upper CI)	Costs attributed to alcohol - Outpatient	Costs attributed to alcohol - Outpatient (Lower CI)	Costs attributed to alcohol - Outpatient (Upper CI)	Costs attributed to alcohol - Absenteeism	Costs attributed to alcohol - Absenteeism (Lower CI)	Costs attributed to alcohol - Absenteeism (Upper CI)
Hypertensive heart disease	136,960.29	55,981.22	262,293.28	36,464.97	14,904.71	69,834.23	317,570.02	129,803.73	608,179.81
Atrial fibrillation and flutter	181,536.10	107,528.77	262,343.52	2,055.68	1,217.64	2,970.73	62,421.30	36,973.83	90,206.98
Cirrhosis and other chronic liver diseases due to alcohol use	5,841,007.70	3,054,896.83	9,331,600.72	111,991.51	58,572.51	178,917.76	1,255,089.15	656,422.33	2,005,131.89
Pancreatitis	918,520.97	273,589.25	2,362,646.40	141,899.63	42,266.01	364,998.35	257,222.36	76,615.86	661,634.86
Epilepsy	986,787.76	455,006.10	1,591,847.39	276,025.48	127,274.87	445,273.50	1,032,196.92	475,944.19	1,665,099.67
Transport injuries	7,004,189.49	1,682,172.25	13,493,687.84	12,176.71	2,924.44	23,458.64	41,013.42	9,850.05	79,013.04
Unintentional injuries	10,009,986.80	2,397,603.40	20,734,450.16	18,083.69	4,331.42	37,458.12	35,459.71	8,493.35	73,450.40
Self-harm	206,423.96	31,155.65	470,159.27	506.06	76.38	1,152.63	7,600.67	1,147.17	17,311.59
Interpersonal violence	1,800,004.64	406,858.40	3,510,575.22	5,911.20	1,336.12	11,528.70	61,030.09	13,794.75	119,027.88
Intracerebral hemorrhage - Male	2,375,186.35	813,586.45	4,177,823.72	57,775.03	10,295.32	101,623.15	325,085.01	111,353.27	571,806.87
Intracerebral hemorrhage - Female	727,382.24	- 374,912.14	1,950,134.46	18,890.94	5,186.40	50,647.19	66,964.69	18,384.78	179,534.41
Alcohol use disorders	40,491,151.88			21,234,033.59			24,645,505.47		
TOTAL	88,011,434.44	16,586,510.52	91,229,853.10	62,271,452.70	23,738,878.71	59,834,047.97	39,467,693.59	7,505,946.96	24,282,085.56
