# Supplementary material for: Direct and indirect costs attributed to alcohol consumption in Brazil, 2010 to 2018
Source: PLoS One. 2022 Oct 25;17(10):e0270115. doi: 10.1371/journal.pone.0270115 (PMC9595536; doi:10.1371/journal.pone.0270115)
Supplement: S5 Table — Costs attributable to alcohol by type of cost and ICD, Brazil, 2014. (PDF) [file pone.0270115.s005.pdf]

S5 Table: Costs attributable to alcohol by type of cost and ICD, Brazil, 2014

ICD-10	Costs attributed to alcohol - Hospital	Costs attributed to alcohol - Hospital (Lower CI)	Costs attributed to alcohol - Hospital (Upper CI)	Costs attributed to alcohol - Outpatient	Costs attributed to alcohol - Outpatient (Lower CI)	Costs attributed to alcohol - Outpatient (Upper CI)	Costs attributed to alcohol - Absenteeism	Costs attributed to alcohol - Absenteeism (Lower CI)	Costs attributed to alcohol - Absenteeism (Upper CI)
Tuberculosis	1,948,694.14	816,115.11	3,670,447.29	45,896.59	19,221.54	86,448.16	3,303,174.10	1,383,372.71	6,221,667.20
Lower respiratory infections	2,888,090.06	366,758.67	9,973,608.98	9,375.87	1,190.64	32,378.24	91,692.27	11,644.01	316,646.23
Esophageal cancer	1,936,388.68	891,838.00	3,134,824.60	2,065,939.91	951,505.12	3,344,555.43	567,708.91	261,468.37	919,065.41
Liver cancer due to alcohol use	268,444.84	16,952.52	648,289.82	67,515.06	4,263.63	163,047.74	83,439.72	5,269.29	201,505.54
Laryngeal cancer	1,022,940.71	230,912.38	2,148,501.47	1,080,690.46	243,948.45	2,269,794.33	321,292.76	72,526.66	674,817.17
Breast cancer	4,435,245.83	2,795,926.04	6,107,096.58	22,321,896.97	14,071,457.46	30,736,059.76	4,749,854.16	2,994,251.38	6,540,295.46
Colon and rectum cancer	3,600,685.57	1,875,578.59	5,399,296.61	6,414,327.05	3,341,189.96	9,618,405.62	1,263,380.33	658,088.31	1,894,462.87
Lip and oral cavity cancer	4,081,390.06	2,245,016.36	6,143,986.62	3,799,382.32	2,089,894.70	5,719,461.69	1,160,004.72	638,074.17	1,746,231.88
Nasopharyngeal cancer	238,503.74	216,607.23	260,671.69	689,162.95	625,892.40	753,217.85	255,081.60	231,663.11	278,790.39
Other pharyngeal cancers	1,146,169.91	631,861.75	1,717,228.23	3,726,889.64	2,054,563.63	5,583,744.63	683,244.16	376,659.56	1,023,658.15
Hypertensive heart disease	141,480.36	57,828.75	270,949.69	48,737.05	19,920.81	93,336.56	222,688.51	91,021.81	426,471.80
Atrial fibrillation and flutter	186,231.70	110,310.09	269,129.27	2,701.42	1,600.12	3,903.91	65,320.97	38,691.38	94,397.38

ICD-10	Costs attributed to alcohol - Hospital	Costs attributed to alcohol - Hospital (Lower CI)	Costs attributed to alcohol - Hospital (Upper CI)	Costs attributed to alcohol - Outpatient	Costs attributed to alcohol - Outpatient (Lower CI)	Costs attributed to alcohol - Outpatient (Upper CI)	Costs attributed to alcohol - Absenteeism	Costs attributed to alcohol - Absenteeism (Lower CI)	Costs attributed to alcohol - Absenteeism (Upper CI)
Cirrhosis and other chronic liver diseases due to alcohol use	6,560,677.75	3,431,290.40	10,481,346.44	90,136.59	47,142.20	144,002.31	1,336,497.00	698,999.33	2,135,189.17
Pancreatitis	934,937.55	278,479.07	2,404,873.60	169,583.15	50,511.78	436,206.71	246,450.82	73,407.46	633,927.97
Epilepsy	1,080,854.85	498,380.27	1,743,592.73	232,284.60	107,106.02	374,712.43	960,679.85	442,967.79	1,549,731.14
Transport injuries	7,407,663.25	1,779,073.17	14,270,986.75	35,844.94	8,608.76	69,055.87	72,817.67	17,488.37	140,284.45
Unintentional injuries	10,649,832.63	2,550,860.00	22,059,811.71	27,087.53	6,488.04	56,108.47	55,347.82	13,256.97	114,646.17
Self-harm	198,444.06	29,951.24	451,983.95	1,014.67	153.14	2,311.05	11,047.96	1,667.47	25,163.26
Interpersonal violence	1,960,988.91	443,245.97	3,824,545.19	7,951.17	1,797.22	15,507.28	89,749.44	20,286.23	175,039.63
Intracerebral hemorrhage - Male	2,188,219.17	749,543.49	3,848,958.60	43,459.95	7,744.42	76,443.70	349,579.09	119,743.37	614,890.62
Intracerebral hemorrhage - Female	670,450.73	- 345,568.13	1,797,499.33	12,040.79	3,305.73	32,281.74	75,430.65	20,709.06	202,231.92
Alcohol use disorders	30,419,525.35			64,198.58			23,544,448.94		
TOTAL	83,965,859.85	19,670,960.98	100,627,629.13	40,956,117.25	23,657,505.77	59,610,983.48	39,508,931.43	8,171,256.82	25,929,113.81
