# Supplementary material for: Direct and indirect costs attributed to alcohol consumption in Brazil, 2010 to 2018
Source: PLoS One. 2022 Oct 25;17(10):e0270115. doi: 10.1371/journal.pone.0270115 (PMC9595536; doi:10.1371/journal.pone.0270115)
Supplement: S6 Table — Costs attributable to alcohol by type of cost and ICD, Brazil, 2015. (PDF) [file pone.0270115.s006.pdf]

S6 Table: Costs attributable to alcohol by type of cost and ICD, Brazil, 2015

ICD-10	Costs attributed to alcohol - Hospital	Costs attributed to alcohol - Hospital (Lower CI)	Costs attributed to alcohol - Hospital (Upper CI)	Costs attributed to alcohol - Outpatient	Costs attributed to alcohol - Outpatient (Lower CI)	Costs attributed to alcohol - Outpatient (Upper CI)	Costs attributed to alcohol - Absenteeism	Costs attributed to alcohol - Absenteeism (Lower CI)	Costs attributed to alcohol - Absenteeism (Upper CI)
Tuberculosis	1,961,431.65	821,449.59	3,694,438.92	41,350.91	17,317.80	77,886.18	2,542,526.07	1,064,812.54	4,788,954.68
Lower respiratory infections	2,613,216.62	331,852.48	9,024,372.57	9,678.33	1,229.05	33,422.74	57,761.97	7,335.19	199,472.75
Esophageal cancer	1,773,998.48	817,046.33	2,871,930.70	1,887,169.54	869,169.26	3,055,143.61	461,223.86	212,424.79	746,676.49
Liver cancer due to alcohol use	263,053.86	16,612.08	635,270.70	73,526.90	4,643.29	177,566.24	79,424.66	5,015.74	191,809.24
Laryngeal cancer	988,537.98	223,146.52	2,076,244.77	968,604.25	218,646.80	2,034,377.57	259,799.48	58,645.54	545,661.69
Breast cancer	4,390,601.00	2,767,782.47	6,045,623.04	20,508,300.91	12,928,188.15	28,238,834.86	4,152,181.43	2,617,485.62	5,717,332.03
Colon and rectum cancer	3,543,103.29	1,845,584.28	5,312,950.89	6,118,914.63	3,187,311.15	9,175,429.07	1,138,221.39	592,893.67	1,706,784.66
Lip and oral cavity cancer	4,121,960.68	2,267,332.70	6,205,060.26	3,333,469.82	1,833,614.08	5,018,092.78	1,042,936.35	573,679.34	1,570,001.12
Nasopharyngeal cancer	219,673.80	199,506.02	240,091.58	617,384.63	560,703.89	674,768.03	195,268.99	177,341.77	213,418.45
Other pharyngeal cancers	1,118,611.65	616,669.40	1,675,939.56	3,211,336.70	1,770,349.06	4,811,326.82	525,375.30	289,629.45	787,133.99
Hypertensive heart disease	133,961.20	54,755.37	256,549.70	50,939.85	20,821.18	97,555.15	167,840.21	68,603.09	321,431.56

ICD-10	Costs attributed to alcohol - Hospital	Costs attributed to alcohol - Hospital (Lower CI)	Costs attributed to alcohol - Hospital (Upper CI)	Costs attributed to alcohol - Outpatient	Costs attributed to alcohol - Outpatient (Lower CI)	Costs attributed to alcohol - Outpatient (Upper CI)	Costs attributed to alcohol - Absenteeism	Costs attributed to alcohol - Absenteeism (Lower CI)	Costs attributed to alcohol - Absenteeism (Upper CI)
Atrial fibrillation and flutter	199,358.60	118,085.51	288,099.37	2,508.15	1,485.64	3,624.60	52,622.59	31,169.79	76,046.55
Cirrhosis and other chronic liver diseases due to alcohol use	6,775,184.86	3,543,479.45	10,824,043.25	76,045.01	39,772.19	121,489.60	943,439.73	493,427.03	1,507,240.42
Pancreatitis	936,656.11	278,990.96	2,409,294.13	174,138.01	51,868.48	447,922.87	178,384.21	53,133.25	458,845.07
Epilepsy	1,102,997.94	508,590.41	1,779,313.10	198,269.72	91,421.82	319,840.95	705,228.83	325,179.77	1,137,647.54
Transport injuries	6,730,877.01	1,616,531.73	12,967,146.78	29,159.28	7,003.09	56,175.85	64,317.49	15,446.91	123,908.72
Unintentional injuries	9,933,115.02	2,379,190.99	20,575,219.78	33,887.90	8,116.87	70,194.59	49,142.15	11,770.58	101,791.88
Self-harm	197,253.65	29,771.57	449,272.61	1,211.89	182.91	2,760.24	5,237.09	790.44	11,928.20
Interpersonal violence	1,751,079.22	395,799.69	3,415,155.26	10,101.87	2,283.35	19,701.83	67,390.77	15,232.46	131,433.19
Intracerebral hemorrhage - Male	2,009,505.91	688,327.80	3,534,611.69	41,427.93	7,382.32	72,869.47	266,829.17	91,398.55	469,338.01
Intracerebral hemorrhage - Female	631,338.71	- 325,408.75	1,692,638.76	12,265.09	3,367.31	32,883.08	59,509.48	16,337.99	159,546.76
Alcohol use disorders	25,074,549.85			44,684.35			17,614,843.86		
TOTAL	76,470,067.08	19,195,096.60	95,973,267.42	37,444,375.67	21,624,877.70	54,541,866.13	30,629,505.07	6,721,753.52	20,966,403.01
