# Supplementary material for: Direct and indirect costs attributed to alcohol consumption in Brazil, 2010 to 2018
Source: PLoS One. 2022 Oct 25;17(10):e0270115. doi: 10.1371/journal.pone.0270115 (PMC9595536; doi:10.1371/journal.pone.0270115)
Supplement: S9 Table — Costs attributable to alcohol by type of cost and ICD, Brazil, 2018. (PDF) [file pone.0270115.s009.pdf]

S9 Table: Costs attributable to alcohol by type of cost and ICD, Brazil, 2018

ICD-10	Costs attributed to alcohol - Hospital	Costs attributed to alcohol - Hospital (Lower CI)	Costs attributed to alcohol - Hospital (Upper CI)	Costs attributed to alcohol - Outpatient	Costs attributed to alcohol - Outpatient (Lower CI)	Costs attributed to alcohol - Outpatient (Upper CI)	Costs attributed to alcohol - Absenteeism	Costs attributed to alcohol - Absenteeism (Lower CI)	Costs attributed to alcohol - Absenteeism (Upper CI)
Tuberculosis	1,815,107.83	760,169.03	3,418,831.86	34,937.58	14,631.89	65,806.39	3,002,493.96	1,257,447.56	5,655,323.52
Lower respiratory infections	2,489,851.86	316,186.39	8,598,349.88	13,619.14	1,729.50	47,031.78	81,131.61	10,302.91	280,176.50
Esophageal cancer	1,584,613.42	729,821.70	2,565,334.73	1,837,128.58	846,122.02	2,974,132.18	750,745.73	345,769.21	1,215,384.19
Liver cancer due to alcohol use	292,656.00	18,481.48	706,759.37	68,665.15	4,336.26	165,825.21	122,467.57	7,733.93	295,757.15
Laryngeal cancer	901,233.94	203,439.04	1,892,878.46	881,754.83	199,041.94	1,851,966.13	356,315.39	80,432.46	748,375.87
Breast cancer	4,623,663.38	2,914,702.22	6,366,537.49	21,180,190.62	13,351,739.41	29,163,991.11	7,352,690.81	4,635,048.54	10,124,262.49
Colon and rectum cancer	3,676,813.21	1,915,233.08	5,513,451.45	6,651,245.45	3,464,599.54	9,973,669.27	1,869,972.39	974,059.00	2,804,059.22
Lip and oral cavity cancer	3,707,499.68	2,039,353.58	5,581,144.68	2,996,743.15	1,648,393.64	4,511,195.83	1,504,356.44	827,488.86	2,264,607.33
Nasopharyngeal cancer	187,950.93	170,695.56	205,420.21	575,504.29	522,668.49	628,995.08	300,290.75	272,721.71	328,201.56
Other pharyngeal cancers	1,110,249.53	612,059.52	1,663,411.17	3,391,563.54	1,869,704.70	5,081,348.40	917,132.88	505,597.98	1,374,077.67
Hypertensive heart disease	100,092.88	40,912.01	191,688.33	29,730.70	12,152.14	56,937.39	191,910.68	78,441.67	367,529.03
Atrial fibrillation and flutter	210,857.51	124,896.63	304,716.81	2,837.33	1,680.63	4,100.32	93,607.13	55,446.05	135,274.60

ICD-10	Costs attributed to alcohol - Hospital	Costs attributed to alcohol - Hospital (Lower CI)	Costs attributed to alcohol - Hospital (Upper CI)	Costs attributed to alcohol - Outpatient	Costs attributed to alcohol - Outpatient (Lower CI)	Costs attributed to alcohol - Outpatient (Upper CI)	Costs attributed to alcohol - Absenteeism	Costs attributed to alcohol - Absenteeism (Lower CI)	Costs attributed to alcohol - Absenteeism (Upper CI)
Cirrhosis and other chronic liver diseases due to alcohol use	6,902,847.33	3,610,248.01	11,027,996.95	68,281.20	35,711.65	109,086.12	1,209,458.52	632,557.12	1,932,232.34
Pancreatitis	906,494.41	270,007.04	2,331,711.33	275,129.23	81,949.57	707,695.42	232,211.18	69,166.07	597,300.36
Epilepsy	1,120,416.04	516,621.87	1,807,411.30	146,704.75	67,645.30	236,658.35	1,114,145.91	513,730.72	1,797,296.57
Transport injuries	6,559,055.27	1,575,265.89	12,636,129.32	33,801.95	8,118.10	65,120.02	88,864.66	21,342.32	171,199.25
Unintentional injuries	10,164,009.74	2,434,495.15	21,053,489.64	44,343.00	10,621.09	91,851.04	68,834.66	16,487.36	142,582.48
Self-harm	190,798.32	28,797.26	434,569.70	1,023.58	154.49	2,331.34	14,642.79	2,210.04	33,350.99
Interpersonal violence	1,666,372.28	376,653.23	3,249,950.09	15,571.16	3,519.58	30,368.66	110,698.74	25,021.44	215,897.37
Intracerebral hemorrhage - Male	1,900,978.37	651,153.22	3,343,717.64	30,765.50	5,482.31	54,114.84	389,205.95	133,316.99	684,592.11
Intracerebral hemorrhage - Female	620,085.74	- 319,608.67	1,662,469.20	8,546.02	2,346.26	22,912.13	92,576.25	25,416.29	248,199.82
Alcohol use disorders	19,759,665.70			97,456.89			19,173,341.66		
TOTAL	70,491,313.36	18,989,583.24	94,555,969.63	38,385,543.64	22,152,348.49	55,845,137.02	39,037,095.69	10,489,738.22	31,415,680.43
