# Supplementary material for: Direct and indirect costs attributed to alcohol consumption in Brazil, 2010 to 2018
Source: PLoS One. 2022 Oct 25;17(10):e0270115. doi: 10.1371/journal.pone.0270115 (PMC9595536; doi:10.1371/journal.pone.0270115)
Supplement: S10 Table — (PDF) [file pone.0270115.s010.pdf]

S10 Table: Relative risk, Prevalence of alcohol intake, Population Attributable Risk by disease or injuries and level of consumption

Disease or injuries (ICD-10)	Level of Consumption	Sex	Relative risk*	CI 95%	Prevalence 2019	PAR	lower PAR	Upper PAR
Tuberculosis	72 g/day	Both	3.5070	(2,596 - 4,474)	0.0118	0.02873	0.01848	0.03938
Tuberculosis	60 g/day	Both	2.9940	(1,972 - 4,204)	0.0057	0.01124	0.00551	0.01794
Tuberculosis	48 g/day	Both	2.5350	(1,701 - 3,510)	0.0105	0.01586	0.00731	0.02568
Tuberculosis	36 g/day	Both	2.0580	(1,485 - 2,795)	0.0283	0.02907	0.01354	0.04834
Tuberculosis	24 g/day	Both	1.5310	(1,165 - 1,980)	0.0606	0.03118	0.00990	0.05606
Tuberculosis	12 g/day	Both	1.1010	(0,815 - 1,425)	0.1470	0.01463	-0.02796	0.05880
Tuberculosis	0 g/day	Both	1.0000	(1,0 - 1,0)	0.7361	0.13071		
Lower respiratory infections	72 g/day	Both	1.3570	(1,113 - 1,648)	0.0118	0.00419	0.00133	0.00759
Lower respiratory infections	60 g/day	Both	1.2260	(1,036 - 1,423)	0.0057	0.00129	0.00021	0.00241
Lower respiratory infections	48 g/day	Both	1.127	(0,936 - 1,327)	0.0105	0.00133	-0.00067	0.00342
Lower respiratory infections	36 g/day	Both	1.064	(0,928 - 1,219)	0.0283	0.00181	-0.00204	0.00616
Lower respiratory infections	24 g/day	Both	1.026	(0,901 - 1,167)	0.0606	0.00157	-0.00604	0.01002
Lower respiratory infections	12 g/day	Both	1.013	(0,951 - 1,084)	0.1470	0.00191	-0.00726	0.01220

Disease or injuries (ICD-10)	Level of Consumption	Sex	Relative risk*	CI 95%	Prevalence 2019	PAR	lower PAR	Upper PAR
Lower respiratory infections	0 g/day	Both	1.0	(1,0 - 1,0)	0.7361	0.01210		
Oesophageal cancer	72 g/day	Both	2.669	(2,074 - 3,348)	0.0118	0.01931	0.01251	0.02696
Esophageal cancer	60 g/day	Both	2.452	(1,905 - 3,094)	0.0057	0.00821	0.00513	0.01180
Esophageal cancer	48 g/day	Both	2.202	(1,730 - 2,703)	0.0105	0.01246	0.00761	0.01757
Esophageal cancer	36 g/day	Both	1.815	(1,468 - 2,222)	0.0283	0.02254	0.01307	0.03343
Esophageal cancer	24 g/day	Both	1.466	(1,209 - 1,764)	0.0606	0.02746	0.01251	0.04425
Esophageal cancer	12 g/day	Both	1.212	(1,031 - 1,439)	0.1470	0.03022	0.00454	0.06062
Esophageal cancer	0 g/day	Both	1.0	(1,0 - 1,0)	0.7361	0.12022		
Liver cancer due to alcohol use	72 g/day	Both	1.424	(1,088 - 1,855)	0.0118	0.00498	0.00104	0.00999
Liver cancer due to alcohol use	60 g/day	Both	1.372	(1,093 - 1,692)	0.0057	0.00212	0.00053	0.00393
Liver cancer due to alcohol use	48 g/day	Both	1.31	(1,036 - 1,639)	0.0105	0.00324	0.00038	0.00666
Liver cancer due to alcohol use	36 g/day	Both	1.225	(1,009 - 1,455)	0.0283	0.00633	0.00025	0.01271
Liver cancer due to alcohol use	24 g/day	Both	1.14	(0,934 - 1,359)	0.0606	0.00841	-0.00402	0.02129
Liver cancer due to alcohol use	12 g/day	Both	1.067	(0,936 - 1,207)	0.1470	0.00975	-0.00950	0.02953
Liver cancer due to alcohol use	0 g/day	Both	1.0	(1,0 - 1,0)	0.7361	0.03483		

Disease or injuries (ICD-10)	Level of Consumption	Sex	Relative risk*	CI 95%	Prevalence 2019	PAR	lower PAR	Upper PAR
Laryngeal cancer	72 g/day	Both	2.461	(1,758 - 3,228)	0.0118	0.01695	0.00887	0.02562
Laryngeal cancer	60 g/day	Both	2.144	(1,460 - 2,935)	0.0057	0.00648	0.00262	0.01091
Laryngeal cancer	48 g/day	Both	1.813	(1,30 - 2,421)	0.0105	0.00846	0.00314	0.01470
Laryngeal cancer	36 g/day	Both	1.531	(1,126 - 2,061)	0.0283	0.01480	0.00355	0.02915
Laryngeal cancer	24 g/day	Both	1.304	(1,006 - 1,659)	0.0606	0.01809	0.00036	0.03840
Laryngeal cancer	12 g/day	Both	1.12	(0,903 - 1,386)	0.1470	0.01733	-0.01447	0.05370
Laryngeal cancer	0 g/day	Both	1.0	(1,0 - 1,0)	0.7361	0.08212		
Breast cancer	72 g/day	Both	1.476	(1,282 - 1,691)	0.0118	0.00559	0.00332	0.00809
Breast cancer	60 g/day	Both	1.452	(1,312 - 1,599)	0.0057	0.00257	0.00178	0.00340
Breast cancer	48 g/day	Both	1.443	(1,348 - 1,542)	0.0105	0.00463	0.00364	0.00566
Breast cancer	36 g/day	Both	1.433	(1,311 - 1,551)	0.0283	0.01211	0.00872	0.01535
Breast cancer	24 g/day	Both	1.329	(1,237 - 1,419)	0.0606	0.01955	0.01416	0.02476
Breast cancer	12 g/day	Both	1.17	(1,081 - 1,265)	0.1470	0.02438	0.01177	0.03749
Breast cancer	0 g/day	Both	1.0	(1,0 - 1,0)	0.7361	0.06882		
Colon and rectum cancer	72 g/day	Both	1.616	(1,38 - 1,861)	0.0118	0.00722	0.00446	0.01006

Disease or injuries (ICD-10)	Level of Consumption	Sex	Relative risk*	CI 95%	Prevalence 2019	PAR	lower PAR	Upper PAR
Colon and rectum cancer	60 g/day	Both	1.468	(1,329 - 1,615)	0.0057	0.00266	0.00187	0.00349
Colon and rectum cancer	48 g/day	Both	1.323	(1,156 - 1,501)	0.0105	0.00338	0.00164	0.00523
Colon and rectum cancer	36 g/day	Both	1.237	(1,148 - 1,336)	0.0283	0.00666	0.00417	0.00942
Colon and rectum cancer	24 g/day	Both	1.156	(1,067 - 1,248)	0.0606	0.00937	0.00404	0.01481
Colon and rectum cancer	12 g/day	Both	1.078	(1,034 - 1,124)	0.1470	0.01134	0.00497	0.01790
Colon and rectum cancer	0 g/day	Both	1.0	(1,0 - 1,0)	0.7361	0.04062		
Lip and oral cavity cancer	72 g/day	Both	4.858	(3,74 - 6,076)	0.0118	0.04354	0.03132	0.05651
Lip and oral cavity cancer	60 g/day	Both	3.766	(2,839 - 4,9)	0.0057	0.01552	0.01037	0.02175
Lip and oral cavity cancer	48 g/day	Both	2.991	(2,283 - 3,896)	0.0105	0.02048	0.01329	0.02951
Lip and oral cavity cancer	36 g/day	Both	2.311	(1,757 - 2,929)	0.0283	0.03577	0.02097	0.05176
Lip and oral cavity cancer	24 g/day	Both	1.738	(1,383 - 2,161)	0.0606	0.04281	0.02268	0.06573
Lip and oral cavity cancer	12 g/day	Both	1.293	(1,076 - 1,551)	0.1470	0.04129	0.01105	0.07493
Lip and oral cavity cancer	0 g/day	Both	1.0	(1,0 - 1,0)	0.7361	0.19942		
Nasopharyngeal cancer	72 g/day	Both	4.545	(4,1 - 4,982)	0.0118	0.04015	0.03529	0.04488
Nasopharyngeal cancer	60 g/day	Both	3.803	(3,509 - 4,102)	0.0057	0.01573	0.01410	0.01737

Disease or injuries (ICD-10)	Level of Consumption	Sex	Relative risk*	CI 95%	Prevalence 2019	PAR	lower PAR	Upper PAR
Nasopharyngeal cancer	48 g/day	Both	3.062	(2,873 - 3,258)	0.0105	0.02119	0.01929	0.02316
Nasopharyngeal cancer	36 g/day	Both	2.385	(2,25 - 2,552)	0.0283	0.03772	0.03417	0.04207
Nasopharyngeal cancer	24 g/day	Both	1.839	(1,77 - 1,907)	0.0606	0.04838	0.04458	0.05210
Nasopharyngeal cancer	12 g/day	Both	1.371	(1,341 - 1,398)	0.1470	0.05172	0.04773	0.05527
Nasopharyngeal cancer	0 g/day	Both	1.0	(1,0 - 1,0)	0.7361	0.21489		
Other pharyngeal cancers	72 g/day	Both	4.764	(3,315 - 6,576)	0.0118	0.04253	0.02659	0.06173
Other pharyngeal cancers	60 g/day	Both	3.972	(2,813 - 5,354)	0.0057	0.01666	0.01023	0.02422
Other pharyngeal cancers	48 g/day	Both	3.199	(2,202 - 4,407)	0.0105	0.02257	0.01246	0.03454
Other pharyngeal cancers	36 g/day	Both	2.519	(1,843 - 3,299)	0.0283	0.04122	0.02330	0.06109
Other pharyngeal cancers	24 g/day	Both	1.943	(1,467 - 2,484)	0.0606	0.05406	0.02752	0.08251
Other pharyngeal cancers	12 g/day	Both	1.472	(1,234 - 1,742)	0.1470	0.06488	0.03325	0.09835
Other pharyngeal cancers	0 g/day	Both	1.0	(1,0 - 1,0)	0.7361	0.24191		
Hypertensive heart disease	72 g/day	Both	1.86	(1,445 - 2,358)	0.0118	0.01005	0.00522	0.01577
Hypertensive heart disease	60 g/day	Both	1.705	(1,297 - 2,175)	0.0057	0.00400	0.00169	0.00665
Hypertensive heart disease	48 g/day	Both	1.614	(1,25 - 2,049)	0.0105	0.00641	0.00262	0.01089

Disease or injuries (ICD-10)	Level of Consumption	Sex	Relative risk*	CI 95%	Prevalence 2019	PAR	lower PAR	Upper PAR
Hypertensive heart disease	36 g/day	Both	1.479	(1,232 - 1,759)	0.0283	0.01337	0.00652	0.02103
Hypertensive heart disease	24 g/day	Both	1.315	(1,136 - 1,526)	0.0606	0.01873	0.00817	0.03089
Hypertensive heart disease	12 g/day	Both	1.046	(0,913 - 1,198)	0.1470	0.00672	-0.01295	0.02828
Hypertensive heart disease	0 g/day	Both	1.0	(1,0 - 1,0)	0.7361	0.05928		
Atrial fibrillation and flutter	72 g/day	Both	1.535	(1,348 - 1,728)	0.0118	0.00627	0.00409	0.00852
Atrial fibrillation and flutter	60 g/day	Both	1.411	(1,26 - 1,569)	0.0057	0.00234	0.00148	0.00323
Atrial fibrillation and flutter	48 g/day	Both	1.312	(1,218 - 1,407)	0.0105	0.00327	0.00228	0.00426
Atrial fibrillation and flutter	36 g/day	Both	1.214	(1,145 - 1,29)	0.0283	0.00602	0.00409	0.00814
Atrial fibrillation and flutter	24 g/day	Both	1.131	(1,067 - 1,204)	0.0606	0.00788	0.00404	0.01221
Atrial fibrillation and flutter	12 g/day	Both	1.066	(1,034 - 1,102)	0.1470	0.00961	0.00497	0.01477
Atrial fibrillation and flutter	0 g/day	Both	1.0	(1,0 - 1,0)	0.7361	0.03538		
Cirrhosis and other chronic liver diseases due to alcohol use	72 g/day	Both	9.427	(6,131 - 13,804)	0.0118	0.09044	0.05709	0.13126
Cirrhosis and other chronic liver diseases due to alcohol use	60 g/day	Both	6.274	(3,958 - 9,319)	0.0057	0.02918	0.01658	0.04527
Cirrhosis and other chronic liver diseases due to alcohol use	48 g/day	Both	4.673	(3,25 - 6,717)	0.0105	0.03713	0.02308	0.05663
Cirrhosis and other chronic liver diseases due to alcohol use	36 g/day	Both	3.274	(2,309 - 4,485)	0.0283	0.06046	0.03572	0.08977

Disease or injuries (ICD-10)	Level of Consumption	Sex	Relative risk*	CI 95%	Prevalence 2019	PAR	lower PAR	Upper PAR
Cirrhosis and other chronic liver diseases due to alcohol use	24 g/day	Both	2.055	(1,521 - 2,688)	0.0606	0.06009	0.03061	0.09280
Cirrhosis and other chronic liver diseases due to alcohol use	12 g/day	Both	1.243	(0,943 - 1,611)	0.1470	0.03449	-0.00845	0.08241
Cirrhosis and other chronic liver diseases due to alcohol use	0 g/day	Both	1.0	(1,0 - 1,0)	0.7361	0.31181		
Pancreatitis	72 g/day	Both	3.298	(2,473 - 4,458)	0.0118	0.02640	0.01708	0.03920
Pancreatitis	60 g/day	Both	2.217	(1,415 - 3,389)	0.0057	0.00689	0.00236	0.01343
Pancreatitis	48 g/day	Both	1.717	(1,199 - 2,477)	0.0105	0.00747	0.00209	0.01527
Pancreatitis	36 g/day	Both	1.471	(1,062 - 2,021)	0.0283	0.01315	0.00175	0.02808
Pancreatitis	24 g/day	Both	1.228	(0,874 - 1,67)	0.0606	0.01363	-0.00769	0.03902
Pancreatitis	12 g/day	Both	1.073	(0,791 - 1,481)	0.1470	0.01062	-0.03170	0.06604
Pancreatitis	0 g/day	Both	1.0	(1,0 - 1,0)	0.7361	0.07816		
Epilepsy	72 g/day	Both	2.48	(1,929 - 3,144)	0.0118	0.01716	0.01084	0.02467
Epilepsy	60 g/day	Both	2.186	(1,781 - 2,622)	0.0057	0.00671	0.00443	0.00916
Epilepsy	48 g/day	Both	1.872	(1,438 - 2,369)	0.0105	0.00907	0.00458	0.01417
Epilepsy	36 g/day	Both	1.585	(1,303 - 1,898)	0.0283	0.01629	0.00850	0.02478
Epilepsy	24 g/day	Both	1.353	(1,118 - 1,633)	0.0606	0.02094	0.00710	0.03694

Disease or injuries (ICD-10)	Level of Consumption	Sex	Relative risk*	CI 95%	Prevalence 2019	PAR	lower PAR	Upper PAR
Epilepsy	12 g/day	Both	1.177	(1,059 - 1,316)	0.1470	0.02536	0.00860	0.04439
Epilepsy	0 g/day	Both	1.0	(1,0 - 1,0)	0.7361	0.09554		
Transport injuries	72 g/day	Both	1.552	(1,201 - 2,032)	0.0118	0.00647	0.00237	0.01203
Transport injuries	60 g/day	Both	1.456	(1,186 - 1,818)	0.0057	0.00259	0.00106	0.00464
Transport injuries	48 g/day	Both	1.366	(1,101 - 1,692)	0.0105	0.00383	0.00106	0.00721
Transport injuries	36 g/day	Both	1.288	(1,089 - 1,534)	0.0283	0.00808	0.00251	0.01489
Transport injuries	24 g/day	Both	1.22	(1,062 - 1,4)	0.0606	0.01316	0.00374	0.02367
Transport injuries	12 g/day	Both	1.163	(1,021 - 1,346)	0.1470	0.02340	0.00308	0.04840
Transport injuries	0 g/day	Both	1.0	(1,0 - 1,0)	0.7361	0.05753		
Unintentional injuries	72 g/day	Both	1.266	(1,063 - 1,555)	0.0118	0.00313	0.00074	0.00651
Unintentional injuries	60 g/day	Both	1.221	(1,059 - 1,46)	0.0057	0.00126	0.00034	0.00262
Unintentional injuries	48 g/day	Both	1.182	(1,024 - 1,428)	0.0105	0.00191	0.00025	0.00447
Unintentional injuries	36 g/day	Both	1.168	(1,054 - 1,347)	0.0283	0.00473	0.00153	0.00972
Unintentional injuries	24 g/day	Both	1.154	(1,046 - 1,319)	0.0606	0.00925	0.00278	0.01896
Unintentional injuries	12 g/day	Both	1.09	(1,016 - 1,187)	0.1470	0.01306	0.00235	0.02675

Disease or injuries (ICD-10)	Level of Consumption	Sex	Relative risk*	CI 95%	Prevalence 2019	PAR	lower PAR	Upper PAR
Unintentional injuries	0 g/day	Both	1.0	(1,0 - 1,0)	0.7361	0.03333		
Self-harm	72 g/day	Both	1.927	(1,398 - 2,665)	0.0118	0.01082	0.00467	0.01927
Self-harm	60 g/day	Both	1.734	(1,29 - 2,308)	0.0057	0.00417	0.00165	0.00740
Self-harm	48 g/day	Both	1.545	(1,132 - 2,048)	0.0105	0.00569	0.00138	0.01088
Self-harm	36 g/day	Both	1.376	(1,05 - 1,751)	0.0283	0.01053	0.00141	0.02081
Self-harm	24 g/day	Both	1.23	(0,972 - 1,533)	0.0606	0.01375	-0.00170	0.03129
Self-harm	12 g/day	Both	1.107	(0,908 - 1,343)	0.1470	0.01549	-0.01371	0.04800
Self-harm	0 g/day	Both	1.0	(1,0 - 1,0)	0.7361	0.06044		
Interpersonal violence	72 g/day	Both	1.516	(1,255 - 1,867)	0.0118	0.00605	0.00300	0.01013
Interpersonal violence	60 g/day	Both	1.452	(1,215 - 1,719)	0.0057	0.00257	0.00122	0.00408
Interpersonal violence	48 g/day	Both	1.396	(1,118 - 1,739)	0.0105	0.00414	0.00124	0.00770
Interpersonal violence	36 g/day	Both	1.345	(1,14 - 1,585)	0.0283	0.00967	0.00395	0.01629
Interpersonal violence	24 g/day	Both	1.256	(1,055 - 1,46)	0.0606	0.01528	0.00332	0.02712
Interpersonal violence	12 g/day	Both	1.129	(0,963 - 1,317)	0.1470	0.01861	-0.00547	0.04452
Interpersonal violence	0 g/day	Both	1.0	(1,0 - 1,0)	0.7361	0.00000		

Disease or injuries (ICD-10)	Level of Consumption	Sex	Relative risk*	CI 95%	Prevalence 2019	PAR	lower PAR	Upper PAR
Intracerebral hemorrhage	72 g/day	Male	1.971	(1,663 - 2,316)	0.0205	0.01952	0.01341	0.02627
Intracerebral hemorrhage	60 g/day	Male	1.705	(1,45 - 1,991)	0.0102	0.00714	0.00457	0.01001
Intracerebral hemorrhage	48 g/day	Male	1.458	(1,182 - 1,1768)	0.0174	0.00791	0.00316	0.00307
Intracerebral hemorrhage	36 g/day	Male	1.31	(1,105 - 1,539)	0.0484	0.01478	0.00506	0.02542
Intracerebral hemorrhage	24 g/day	Male	1.162	(0,973 - 1,358)	0.0940	0.01500	-0.00254	0.03256
Intracerebral hemorrhage	12 g/day	Male	1.068	(0,945 - 1,214)	0.1804	0.01212	-0.01002	0.03717
Intracerebral hemorrhage	0 g/day	Male	1.0	(1,0 - 1,0)	0.6291	0.07646		
Intracerebral hemorrhage	72 g/day	Female	2.276	(1,701 - 2,934)	0.0041	0.00520	0.00287	0.00787
Intracerebral hemorrhage	60 g/day	Female	1.964	(1,536 - 2,464)	0.0017	0.00164	0.00091	0.00248
Intracerebral hemorrhage	48 g/day	Female	1.614	(1,245 - 2,048)	0.0043	0.00263	0.00105	0.00449
Intracerebral hemorrhage	36 g/day	Female	1.337	(1,065 - 1,664)	0.0107	0.00359	0.00070	0.00705
Intracerebral hemorrhage	24 g/day	Female	1.11	(0,884 - 1,367)	0.0312	0.00342	-0.00363	0.01132
Intracerebral hemorrhage	12 g/day	Female	1.031	(0,897 - 1,18)	0.1176	0.00363	-0.01226	0.02073
Intracerebral hemorrhage	0 g/day	Female	1.0	(1,0 - 1,0)	0.8304	0.02012		

Disease or injuries (ICD-10)	Level of Consumption	Sex	Relative risk*	CI 95%	Prevalence 2019	PAR	lower PAR	Upper PAR

* Data of relative risk are from Global Burden Disease 2017. Reference: Stanaway, J. D. et al. Global, regional, and national comparative risk assessment of 84 behavioural, environmental and occupational, and metabolic risks or clusters of risks for 195 countries and territories, 1990-2017: A systematic analysis for the Global Burden of Disease Study 2017. Lancet 392, (2018)
